# The role of hepatocyte growth factor in mesenchymal stem cell-induced recovery in spinal cord injured rats

**DOI:** 10.1186/s13287-020-01691-x

**Published:** 2020-05-14

**Authors:** Peiwen Song, Tianyu Han, Xia Xiang, Ying Wang, Huang Fang, Yang Niu, Cailiang Shen

**Affiliations:** 1 Department of Orthopedics (Spinal Surgery), The First Affiliated Hospital of Anhui Medical University, 218 Jixi Road, Shushan District, Hefei City, Anhui Province China; 2 Department of Radiology, The First Affiliated Hospital of Anhui Medical University, Hefei, China; 3 Department of Spinal Surgery, The First Affiliated Hospital of USTC, Hefei, China

**Keywords:** Neural stem cells, Mesenchymal stem cells, Bone morphogenetic proteins, Hepatocyte growth factor, Inflammation

## Abstract

**Background:**

Mesenchymal stem cells (MSCs) have become a promising treatment for spinal cord injury (SCI) due to the fact that they provide a favorable environment. Treatment using MSCs results in a better neurological functional improvement through the promotion of nerve cell regeneration and the modulation of inflammation. Many studies have highlighted that the beneficial effects of MSCs are more likely associated with their secreted factors. However, the identity of the factor that plays a key role in the MSC-induced neurological functional recovery following SCI as well as its molecular mechanism still remains unclear.

**Methods:**

A conditioned medium (collected from the MSCs) and hepatocyte growth factor (HGF) were used to test the effects on the differentiation of neural stem cells (NSCS) in the presence of BMP4 with or without a c-Met antibody. In SCI rats, Western blot, ELISA, immunohistochemistry, and hematoxylin-eosin staining were used to investigate the biological effects of MSC-conditioned medium and HGF on nerve cell regeneration and inflammation with or without the pre-treatment using a c-Met antibody. In addition, the possible molecular mechanism (cross-talk between HGF/c-Met and the BMP/Smad 1/5/8 signaling pathway) was also detected by Western blot both in vivo and in vitro.

**Results:**

The conditioned medium from bone marrow-derived MSCs (BMSCs) was able to promote the NSC differentiation into neurons in vitro and the neurite outgrowth in the scar boundary of SCI rats by inhibiting the BMP/Smad signaling pathway as well as reduces the secondary damage through the modulation of the inflammatory process. The supplementation of HGF showed similar biological effects to those of BMSC-CM, whereas a functional blocking of the c-Met antibody or HGF knockdown in BMSCs significantly reversed the functional improvement mediated by the BMSC-CM.

**Conclusions:**

The MSC-associated biological effects on the recovery of SCI rats mainly depend on the secretion of HGF.

## Introduction

A spinal cord injury (SCI) is a fatal neurological damage, often causing an incomplete or complete loss of neural function [1, 2]. Although the primary damage is directly associated with severe spinal cord impairment, the pathophysiology following the primary damage, which is also known as the secondary damage, seems to be more closely associated with the final outcome of neurological function [3, 4]. Inflammation has been proven to play a critical role in such secondary damage following SCI. After the trauma, several pro-inflammatory cytokines, triggers of post-trauma-induced inflammation, accumulate in the injured lesion, causing the recruitment and activation of inflammatory cells [5, 6]. At the same time, the injured lesion is not able to produce a sufficient amount of anti-inflammatory cytokines in comparison to the marked increased in pro-inflammatory cytokines, leading to an inflammatory cytokine imbalance [6, 7]. This imbalance results during over-reactive inflammation, which causes the loss of surviving nerve cells and poor functional outcomes. Thus, the use of anti-inflammatory drugs to suppress inflammation after SCI has been proven to produce beneficial effects with regard to the prevention of extensive tissue loss and achieve better neurological function outcomes [8–10].

Environmental factors or molecules have also been shown to influence neurological outcomes following SCI [11, 12]. Numerous studies have found that treating SCI rats with the appropriate neurotrophins will promote the regeneration of neurons and injured axon regrowth [13–15]. However, the severe environment following SCI usually causes the increased expression of growth-inhibition factors, which are closely associated with the formation of scars. Bone morphogenetic proteins (BMPs) are one of these inhibition factors. BMPs are known to be a member of the transforming growth factor-b superfamily. They are secreted factors and are closely associated with the differentiation of NSCs in the central nervous system (CNS) [16, 17]. Previous studies have demonstrated that, in the spinal cord injury models, BMP expression is upregulated at the site of the damaged lesion [18–20]. Moreover, this upregulation of BMPs has been considered to contribute to the direction of endogenous neural stem cell (ENSC) differentiation toward astrocytes, resulting in a majority generation of astrocytes and a low extent expression of neurons following SCI. Blocking BMP activity helps promote neuron and axon regrowth [21, 22].

Therefore, many studies have focused on the mediation of the post-trauma inflammatory process and BMP expression following the onset of a SCI. One of the most promising studies in this regard involved the transplantation of stem cells, including mesenchymal stem cells (MSCs). Mesenchymal stem cells (MSCs) were first demonstrated and isolated from the bone marrow [23]. Due to their multipotent and self-renewing ability, these cells could be a potential therapy for many diseases. In the research on spinal cord injuries, the transplantation of MSCs has been favored by some researchers because these cells were first thought to generate the three main types of nerve cells (neurons, astrocytes, and oligodendrocytes) needed to replace the damaged cells. However, an increasing amount of studies have found that this neurological improvement might not be caused by the neuronal differentiation of MSCs [24, 25], but through generating a favorable environment for protecting ENSCs from inflammatory damage [26] and promoting their neural differentiation [27–30]. In addition, studies have shown that these beneficial effects of MSCs are most likely due to their secreted factors. Using a conditioned medium (CM), comprised of the BMSC-released factors, a similar beneficial impact as that of BMSCs has been achieved in many different animal models, including SCI rats [30–33]. However, the identity of the cytokine that plays a key role in this MSC-induced neurological functional recovery following SCI and its molecular mechanism still remain unclear.

Hepatocyte growth factor (HGF) was first described as a blood-derived pleiotropic cytokine for hepatocytes [34]. Recent studies have found that HGF and its tyrosine kinase transmembrane receptor c-Met are also expressed in the CNS. In addition, the biological effects of HGF are similar to those of MSCs under many different conditions [35–37]. Therefore, we hypothesized that the way in which the BMSC exerts its biological effect in the CNS might depend on the secretion of HGF. In this study, we prove the key role played by HGF in the functional recovery stimulated by the MSC-conditioned medium (MSC-CM). Bone marrow mesenchymal stem cells (BMSCs) were able to promote endogenous NSC (ENSC) differentiation into neurons by inhibiting the BMP/Smad signaling pathway and suppressing the post-trauma inflammatory process to protect surviving cells in the injured lesions. The MSC-CM treatment significantly improved the neurological the recovery in SCI rats. Moreover, the biological effect of the BMSC-CM can be reversed via treatment with function-blocking c-Met. All these results indicated that MSCs or HGF play a critical role in generating a favorable environment for neurological functional recovery following SCI.

## Methods

### Isolation and cultivation of NSCs

The culturing and isolation of NSCs mainly followed our previous study [38]. The NSCs were isolated from the subventricular zone of SD rats and were suspended as neurospheres in DMEM/F12 (Gibco, USA) containing 2% B27 (Gibco, USA), 10 ng/mL basic fibroblast growth factor (bFGF) (Gibco, USA), and 20 ng/mL epidermal growth factor (EGF) (Gibco, USA). To achieve passage 2, the primary neurospheres were cultured for 7 days and then centrifuged at 800*g* for 5 min. They were then re-suspended in a culture medium (containing DMEM/F12, B27, EGF, and bFGF).

### Mesenchymal stem cell culture, transfection, and the preparation of the conditioned medium

The mesenchymal stem cells were isolated from the bone marrow based on previous research [38]. Briefly, Fischer 344 rats (3–4 months old) were used to harvest the MSCs. The harvested cells were maintained in DMEM (low glucose, Hyclone), supplemented with 1% antibiotic solution and 10% fetal bovine serum (FBS) (Gibco, USA), at a density of 1 × 10^6^ cells/cm^2^ for 1 day. Then, the medium and the non-adherent cells were removed. Adherent cells were washed with phosphate-buffered saline (PBS, Hyclone) twice and re-incubated with 10% FBS-DMEM (low glucose) until 90% of confluence was reached. 0.25% Trypsin (Gibco, USA) was used to harvest the adherent cells and reseeded in 10% FBS-DMEM (low glucose) at a density of 8000 cells/cm^2^ with a medium change every 3 days. The cells were passaged when 90% of confluence was reached.

One hundred millimolars HGF siRNA (AAACTACTGTCGAAATCCTCGAG) was transfected into the passage 3 BMSCs using Lipofectamine 2000 (Invitrogen) for 24 h to knock down HGF. The non-targeting siRNA served as a control. Western blot was used to investigate the effects of HGF knockdown on the BMSCs.

Ninety percent confluent P3 BMSCs were prepared for the collection of the conditioned medium (CM). The cells were washed with PBS three times and cultured for 48 h with a serum-free DMEM/F12 (Gibco, USA) medium. Then, the culture medium was collected as a primary BMSC-conditioned medium. The primary conditioned medium was pooled from different flasks and concentrated using 10 kDa MW filter units (Millipore, USA) at 4000*g* centrifugation for 15 min. The collected CM was filtered by a 0.22-mm filter (Millipore, USA) and stored at − 80 °C.

### Co-culture of NSCs with BMP4 alone or BMP4 in the presence of BMSC-CM or HGF or BMSC-CM + c-Met antibodies or HGF + c-Met antibodies

Passage 2 NSCs were dissociated and seeded on glass coverslips at a density of 1000 cells/cm^2^ in 5% FBS-DMEM/F12 for 24 h. Then, the cells were re-cultured in 5% FBS-DMEM/F12 supplement with 20 ng/ml BMP4 alone or 20 ng/ml BMP with the addition of 1.5 ml of BMSC-CM or HGF (10 ng/mL, 20 ng/mL, 40 ng/mL, R&D Systems) or 1.5 ml BMSC-CM + 100 ng/mL c-Met antibodies (E7050, Med Chen Express) or 40 ng/mL HGF + 100 ng/mL c-Met antibodies or 1.5 ml CM^HGF-siRNA^. The medium was changed at day three. The cells, which were co-cultured for 7 days, were processed for immunohistochemistry. For each culture condition, we randomly selected 10–15 fields, containing a total of 500–1000 cells. Two different blinded individuals quantitated the positive cells in these fields, and the number or proportion of total count was averaged.

### ELISA

ELISA kits (Invitrogen, USA) were used to test the levels of HGF, TNF-α, IL-4, IL-6, and IL-10 following the instructions of the manufacturer. Briefly, after preparing the samples and constructing a standard curve, biotinylated antibodies, a streptavidin-HRP reagent, and a TMB substrate were added to wells, in turn, at RT. A VERSA max microplate reader was used to measure absorbance and calculate the results.

### Experimental spinal cord injury in rats and BMSC-CM administration

Adult (6–8 weeks) female Wistar rats (weight, 200 to 250 g) from the Animal Facility of Anhui Medical University were used and randomly classified into SCI (control), HGF-treated, BMSC-CM-treated, HGF + c-Met antibody-treated, BMSC-CM + c-Met antibody-treated, and CM^HGF-siRNA^-treated groups. The rats were handled in accordance with the guide and welfare for laboratory animals approved by the Ethical Commission. Sample sizes were determined via power analysis. The animals were thoroughly anesthetized via intraperitoneal injection with ketamine (60 mg/kg; WDT, Garbsen) and xylazine (5 mg/kg; WDT, Garbsen). At the T10 level, a laminectomy was performed exposing the dura. After stabilization of the spinal column, a weight-drop injury was induced using Infinite Horizons Spinal Cord Impactor IH-0400. A catheter, inserted under the dura of the injured lesion site, was connected to a mini-osmotic pump (Alzet 1007D, USA) filled with a control medium (DMFM/F12) or BMSC-CM or HGF or HGF + c-Met antibodies or BMSC-CM + c-Met antibodies or CM^HGF-siRNA^. The pump was placed under the rats’ back skin at an infusion speed of 0.5 μl/h and was removed after 1 week. The Basso, Beattie, and Bresnahan (BBB) open-field test was performed blindly independent to evaluate the low extremity motor function of the rats [39] at different time points (day 1, day 4, day 7, day 14, day 17, day 21, day 24, and day 28) for each group. This study was approved by the Ethics Committee of Anhui Medical University (PERMIT 10/16), which follows the guidelines of the Declaration of Helsinki (as revised in Edinburgh 2000).

### Tissue processing, immunohistochemistry, and hematoxylin-eosin staining

After 4 weeks, the animals had been sacrificed, and their spinal cords were extracted and fixed with a solution of 4% paraformaldehyde and 30% sucrose. A 3-mm length of spinal cord tissue (center of the epicenter of the injured lesion) was cut into 35-μm-thick sections by an instrument (RM2135, Leica) and processed for immunohistochemistry, as described in detail [22]. The primary antibodies were rabbit anti-GFAP for astroglia (1:1000) and mouse anti-Map-2 for neurons (APC; 1:500) (Calbiochem, Germany). The secondary antibodies were Alexa Fluor 488 (green, 1:1000) (Molecular Probes, Germany) and Cy5 (red, 1:500) (Dianova, Germany). The sections were observed and photographed using a DM-6B fluorescent microscope (Leica, Germany) connected to a computer screen. The images were analyzed by ImageJ software. For the analysis of the total cavity volume in the injured lesion, the spinal cord was cut into 20-μm-thick cross-sections 4 weeks after the SCI. With hematoxylin-eosin staining, an investigator blindly calculated the volume of the cavity (details of this method were described as previous studies) [40, 41].

### Western blot assay

The cells were washed by PBS three times and reverted in a lysis buffer supplemented with a protease inhibitor cocktail and phosphatase inhibitor cocktail. A 20-mm injured spinal cord tissue (containing the injured epicenter) was lysed in RIPA+PMSF (RIPA to PMSF = 100:1) buffer on ice. The supernatants were collected following sample centrifugation at 4 °C. Bradford protein assay kits (Bio-Rad Laboratories, USA) were used to test the protein concentration.

The protein was separated by sodium dodecyl sulfate-polyacrylamide gel electrophoresis (SDS-PAGE) and transferred to a polyvinylidene fluoride (PVDF) membrane (Millipore, USA). The member was blocked by 5% non-fat milk supplemented with Tween-20 and TBS at room temperature. The blocked membranes were incubated with primary antibodies overnight at 4 °C: Map-2 (1:2000, Abcam, UK), GFAP (1:2000, Abcam, UK), Smad1/5/8 (1:1000, Signaling Technology, USA), and HGF (1:1000, Invitrogen, USA). The membranes were washed with TBST and incubated with secondary antibodies at a concentration of 1:2000 (Santa Cruz Biotechnology, USA) for 1 h at room temperature. The bands were observed with the addition of a SuperSignal West Pico chemiluminescence substrate (Thermo Scientific, USA) and quantified using ImageJ software.

### Statistical analysis

All results were presented as mean ± standard error of the mean. Statistical analysis was conducted using SPSS 16.0 software (Chicago, IL, USA). Student’s *t* test (two groups) or one-way analysis of variance (ANOVA) (more than two groups) with Tukey’s post hoc test was used for comparisons. *P* values < 0.05 were considered statistically significant.

## Results

### BMSC-CM counter-effects of BMP4 on NSCs

**Fig. 1:**
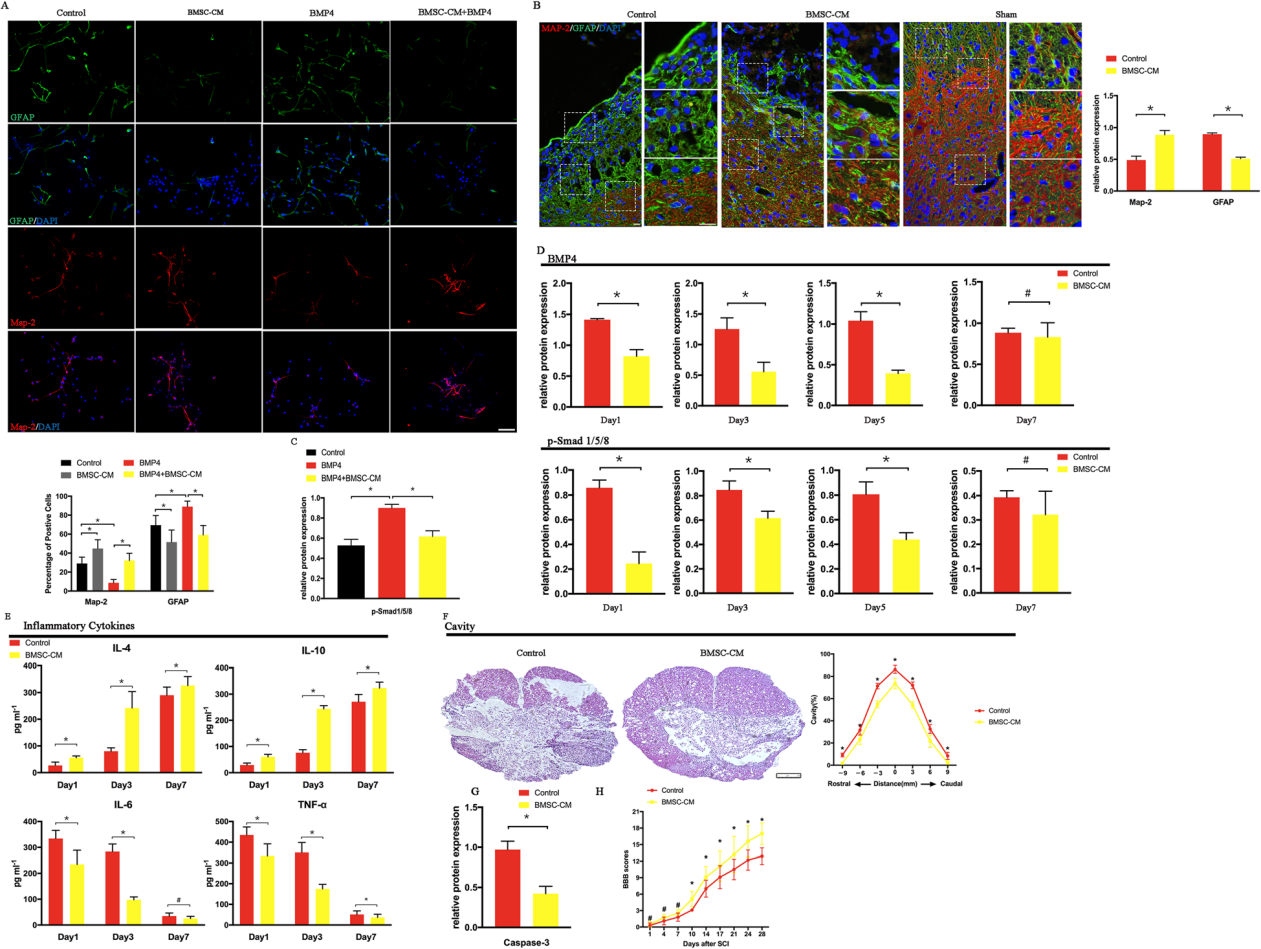
BMSC-CM promotes the functional recovery of SCI rats through the modulation of NSC differentiation and the inhabitation of inflammation. **a** BMSC-CM reduced the proportion of GFAP^+^ cells and increased the percentage of neurons after 7 days co-culturing with NSCs in the presence of BMP4 (20 ng/ml) when compared to NSCs only treated with BMP4 (*n* = 5). **b** In the control groups (SCI rats treated with DMEM/F12), a scar boundary comprised of several GFAP-positive astrocytes was surrounded the cavity in the injured lesion at day 28 after SCI onset. There were few Map-2^+^ cells in this boundary. In contrast, the BMSC-CM-treated rats showed clear neurite outgrowth and extension into the scar boundary around the cavity. Western-blot results also proved this alteration. The SCI rats that received BMSC-CM had a higher Map-2 expression and a lower GFAP expression compared to the rats without BMSC-CM treatment (*n* = 3) (the rats that received sham-operation served as sham groups). **c** The BMP4-induced upregulation of p-Smad 1/5/8 expression was reversed by the treatment of BMSC-CM in the NSCs at day 3 (*n* = 3). **d** Both BMP4 and p-Smad 1/5/8 expressions were downregulated by the treatment of BMSC-CM during the early phases following SCI onset (*n* = 3). **e** Treatment with BMSC-CM markedly decreased pro-inflammatory cytokine expression (TNF-α, IL-6) and increased anti-inflammatory cytokine expression (IL-4, IL-10) in the SCI rats (*n* = 5). Smaller volume of cavity (detected at day 28, *n* = 3, **f**), decreased caspase-3 expression (analysis at day 3, *n* = 3, **g**) and better outcome of neurological scores (tested from day 1 to day 28, *n* = 12, **h**) were found in the rats that received the BMSC-CM treatment. *p < 0.05, ^#^*p* > 0.05; error bars, s.d; scale bars, 100 μm in **a**, **b**; 250 μm in **f**; Western-blot bands revealed in Fig. 4

Based on previous studies, BMPs were considered to be one of the critical factors promoting the differentiation of NSCs into astrocytes following spinal cord injury [18, 21, 22]. To determine whether the BMSC-CM was able to negate the effects of BMP4 on NSCs, we first assayed the effects of BMP4 on NSCs. The exposure of NSCs to 20 ng/ml BMP4 for 7 days resulted in an increase in the glial fibrillary acidic protein (GFAP)-positive astrocytes and a decrease in the microtubule-associated protein 2 (Map-2)-positive neurons (Fig. 1a). In the control groups, approximately 69% of the cells were GFAP-positive astrocytes, while 29% were Map-2^+^ neurons (Fig. 1a). In contrast, after the treatment of BMP4 was administered to the NSCs, the proportion of astrocytes increased to 88%, with a significant reduction in neurons (9%) (Fig. 1a). Then, we analyzed the expression of GFAP and Map-2 after the co-culture of BMSC-CM and BMP4 with NSCs for 7 days. The results showed that the percentage of GFAP- positive cells decreased markedly from 88 to 60%, and the percentage of Map-2-positive neurons increased from 9 to 32% (Fig. 1a).

Next, we detected whether the treatment of the BMSC-CM can increase neurons and reduce scar formation following SCI (Fig. 1b). In the control rats, a scar boundary consisting of several GFAP-positive astrocytes was formed around the cavity at day 28 post-SCI onset. In contrast, the BMSC-CM-treated rats showed clear neurite outgrowth and extension into the scar boundary around the cavity. Western blot also confirmed that the rats receiving BMSC-CM treatment exhibited higher Map-2 expression and lower GFAP expression when compared to the untreated rats (Figs. 1b and 4a). All these results suggested that the BMSC-CM was able to abolish the BMP-induced effect in NSC differentiation.

### BMSC-CM modulated the differentiation of NSCs via the BMP/Smad signaling pathway

To investigate the molecular mechanisms of BMP and BMSC-CM on the NSCs, we evaluated the expression of the BMP4 down-streaming proteins —p-Smad 1/5/8 on day 1 after co-culturing. The addition of BMP4 to the NSCs led to an increased expression of p-Smad1/5/8, indicating that the p-Smad 1/5/8 was upregulated by the BMP4 (Figs. 1c and 4b). This increasing expression was abolished with the treatment of BMSC-CM (Figs. 1c and 4b). In addition, we also detected both BMP4 and p-Smad 1/5/8 during the early phases of the SCI (Figs. 1d and 4c). The results revealed that both BMP4 and p-Smad 1/5/8 expression were reduced with the treatment of BMSC-CM at days 1, 3, and 5 after SCI onset while no difference was noted at day 7. The results also suggested that the BMP/Smad 1/5/8 signaling pathway was inhibited by the BMSC-CM treatment both in vitro and in vivo.

### By mediating the post-trauma inflammatory process, BMSC-CM reduced apoptosis and cavity volume at the injured spinal cord lesion site

Previous studies have pointed out that the inflammation following a SCI can be suppressed by the treatment of BMSC-CM in different models [42–44]. To investigate the effects of BMSC-CM on mediating the post-trauma inflammatory process, we first examined the levels of both the pro-inflammatory (TNF-α and IL-6) cytokines and anti-inflammatory cytokines (IL-4 and IL-10) by ELISA at days 1, 3, and 7 after SCI onset (Fig. 1e). This showed that the expression of IL-6 and TNF-α increased rapidly and peaked at day 1 after SCI onset. In addition, the levels of IL-4 and IL-10 remained quite low level until 7 days after the onset of the SCI. The BMSC-CM addition markedly reduced TNF-α and IL-6 expression. Moreover, a rapid increase in IL-4 and IL-10 expression was found as early as day 3, compared to day 7 in the SCI rats without the BMSC-CM treatment. All these results showed that BMSCM-CM was able to mediate the alleviation of inflammation following SCI.

Then, we evaluated apoptosis at day 3 and the volume of the cavity at day 28 following SCI onset, both of which were considered to be associated with the extent of the post-trauma inflammatory process. By analyzing the expression of caspase-3, the results revealed that it was decreased in the rats (Figs. 1g and 4d) that received the BMSC-CM treatment, indicating that BMSC-CM was able to protect the cells from apoptosis. In addition, the volume of the cavity also decreased with the BMSC-CM addition (Fig. 1f), supporting the hypothesis that the damage was alleviated by this treatment.

As expected, the neurological function was improved by the BMSC-CM treatment. The rats receiving the BMSC-CM treatment exhibited a higher BBB score as early as 14 days after SCI onset and achieved a significant neurological functional improvement over 4 weeks when compared to the control groups (Fig. 1h).

### HGF exerts a similar biological effect as that of the BMSC-CM in treating SCI rats

**Fig. 2:**
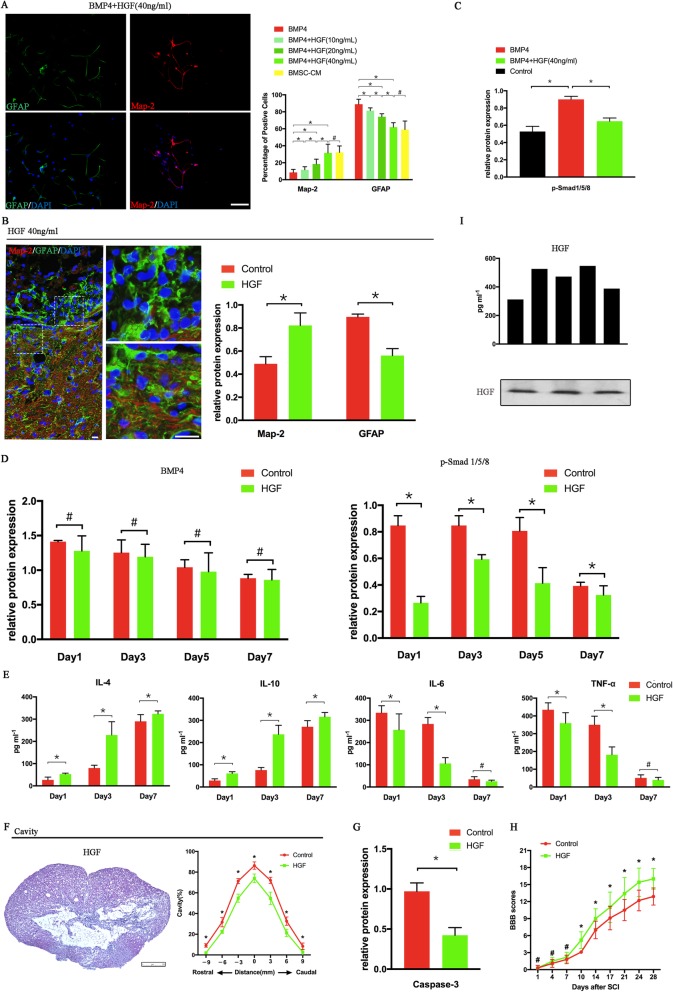
HGF exerted similar effects as BMSC-CM in SCI rats. **a** HGF was able to diminish the effects of BMP4 on the differentiation of NSCs after 7 days of co-culturing. This effect is concentration dependent (*n* = 5). **b** A thinner scar boundary and neurite outgrowth was observed in the rats that received HGF. Higher Map-2 expression and lower GFAP expression in the injured lesion (proven by Western blot) were found when compared to the control rats (SCI rats treated with DMEM/F12) (*n* = 3). **c** P-Smad 1/5/8 expression was inhibited by the treatment of HGF in the NSCs co-cultured with 20 ng/ml BMP4 (*n* = 3). **d** In the SCI rats, the treatment of HGF reduced the p-Smad 1/5/8 expression during the early phases following the onset of SCI (*n* = 3). Meanwhile, the alteration to the BMP4 expression was only observed at day 5 (*n* = 3). Through the downregulation of pro-inflammatory cytokine expression and the upregulation anti-inflammatory cytokine expression (**e**, *n* = 5), HGF decreased the cavity volume (**f**, *n* = 3) and Caspase-3 expression (**g**, *n* = 3) and promoted functional recovery (**h**, *n* = 12) in the SCI rats. **i** ELISA (*n* = 5) and Western blot (*n* = 3) confirmed that BMSC-CM was able to secret HGF in all samples. *p < 0.05, ^#^*p* > 0.05; error bars, s.d; scale bars, 100 μm in **a**, **b**; 250 μm in **f**; Western-blot bands revealed in Fig. 4; the data of cell counting, Western blot, immunohistochemistry, hematoxylin-eosin staining, cavity volume, and BBB scores in the control rats are also revealed in Fig. 1

To determine whether HGF has a similar biological effect as that of BMSC-CM, we first cultured the neurosphere with HGF at different concentrations (10, 20, and 40 ng/ml) in the presence of BMP4 for 7 days (Fig. 2a). With the increasing concentration of HGF, the proportion of neurons increased, while the proportion of astrocytes reduced after 7 days of co-culturing. In the rats that received the HGF (40 ng/ml) treatment, the Map-2 expression increased with the reduction in the GFAP expression. Consistent with the BMSC-CM-induced alteration in the injured lesions, a significant neurite outgrowth and extension along the border of the cavity was also observed in these HGF-treated rats (Fig. 2b).

Next, we identified whether HGF was able to promote the differentiation of NSCs into neurons through inhibiting the Smad proteins. In vitro, the increasing BMP4-associated expression of p-Smad 1/5/8 was reversed by the addition of HGF to the NSCs (Figs. 2c and 4b). In vivo, the treatment of HGF also reduced the p-Smad 1/5/8 expression during the early phases following SCI onset (Figs. 2d and 4c). No alteration in the BMP4 expression was observed (Figs. 2d and 4c).

At last, we evaluated the effect of HGF on the post-trauma inflammatory process. The rats that received the treatment of HGF showed lower levels of TNF-α and IL-6 and higher levels of IL-4 and IL-10 (Fig. 2e). In addition, the apoptosis of the nerve cells (Figs. 2g and 4d) and the volume of the cavity were largely reduced by the treatment with HGF (Fig. 2f). As expected, with the higher proportion of neurons and the inhibition of the inflammatory response, the HGF-treated rats exhibited a higher BBB score when compared to the untreated rats (Fig. 2h).

In summary, HGF was able to promote neuron regeneration and outgrowth by inhibiting the BMP/Smad signaling pathway as well as alleviate the secondary damage after SCI onset by mediating the post-trauma inflammatory response. This suggested that HGF has a similar biological effect to that of BMSC-CM in improving neurological function in SCI rats.

### BMSC-CM exerts its biological effect through c-Met

Based on previous studies and the results above, we considered that HGF might be a candidate factor by which BMSC-CM exerts its biological effect. To identify this hypothesis, we investigated whether BMSCs are able to secrete HGF. ELISA assays of BMSC-CM identified HGF at concentrations ranging from 312 to 547 pg/ml, with an average concentration of approximately 450 (449 ± 98) pg/ml in all samples (Fig. 2i, *n* = 5). Western blot also proved the expression of HGF in BMSC-CM (Fig. 2i, *n* = 3).

**Fig. 3:**
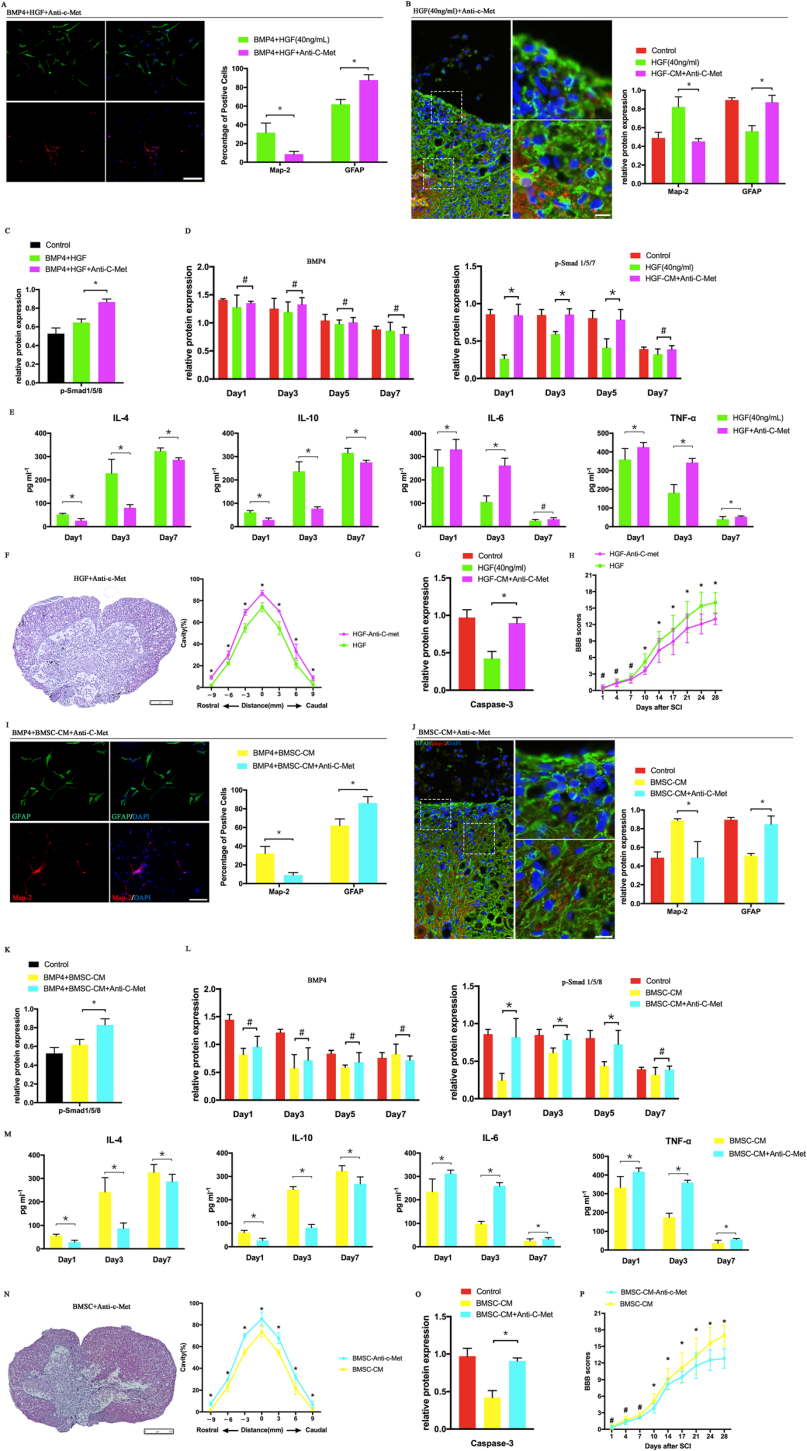
The biological effects of BMSC-CM and HGF were diminished with a function-blocking c-Met antibody. **a**, **i** The pre-treatment of c-Met antibodies reduced the proportion of neurons and increased the proportion of astrocytes in the presence of BMSC-CM or HGF. **b**, **j** The SCI rats, that received c-Met antibody treatment, lost the neurite outgrowth and exhibited a large scar boundary formed around the cavity with the BMSC-CM or HGF treatments (SCI rats treated with DMEM/F12 served as the control and revealed in Fig. 1b). **c**, **k** In vitro, the mediation of BMSC-CM and HGF on the p-Smad 1/5/8 signaling pathway was mainly reversed by the pretreatment with c-Met antibodies (*n* = 3). **d**, **l** The p-Smad 1/5/8 expressions in the SCI rats increased with the treatment of the c-Met antibodies when compared to the BMSC-CM- or HGF-alone-treated rats (*n* = 3). Meanwhile, the BMP4 expressions were not altered by the treatment of c-Met antibodies in the BMSC-CM- or HGF-treated SCI rats (*n* = 3). **e**, **m** The BMSCM-CM and HGF lost their effect on mediating the inflammation in SCI rats with the treatment of the c-Met antibodies (*n* = 3). The cavity volume (**f**, **n**, *n* = 3) and Caspase-3 expression (**g**, **o**, *n* = 3) increased with worsening BBB scores (**h**, **p**, *n* = 12) in c-Met antibody and BMSC-CM/HGF co-treatment rats when compared to the BMSC/CM- or HGF-treated rats. *p < 0.05, ^#^*p* > 0.05; error bars, s.d; scale bars, 100 μm in **a**, **b**, **i**, **j**; 250 μm in **f**, **n**; Western blot bands are revealed in Fig. 4; the data of cell counting, Western blot, immunohistochemistry, hematoxylin-eosin staining, cavity volume and BBB scores in the BMSC-CM-treated and HGF-treated rats are also revealed in Figs. 1 and 2, respectively

**Fig. 4:**
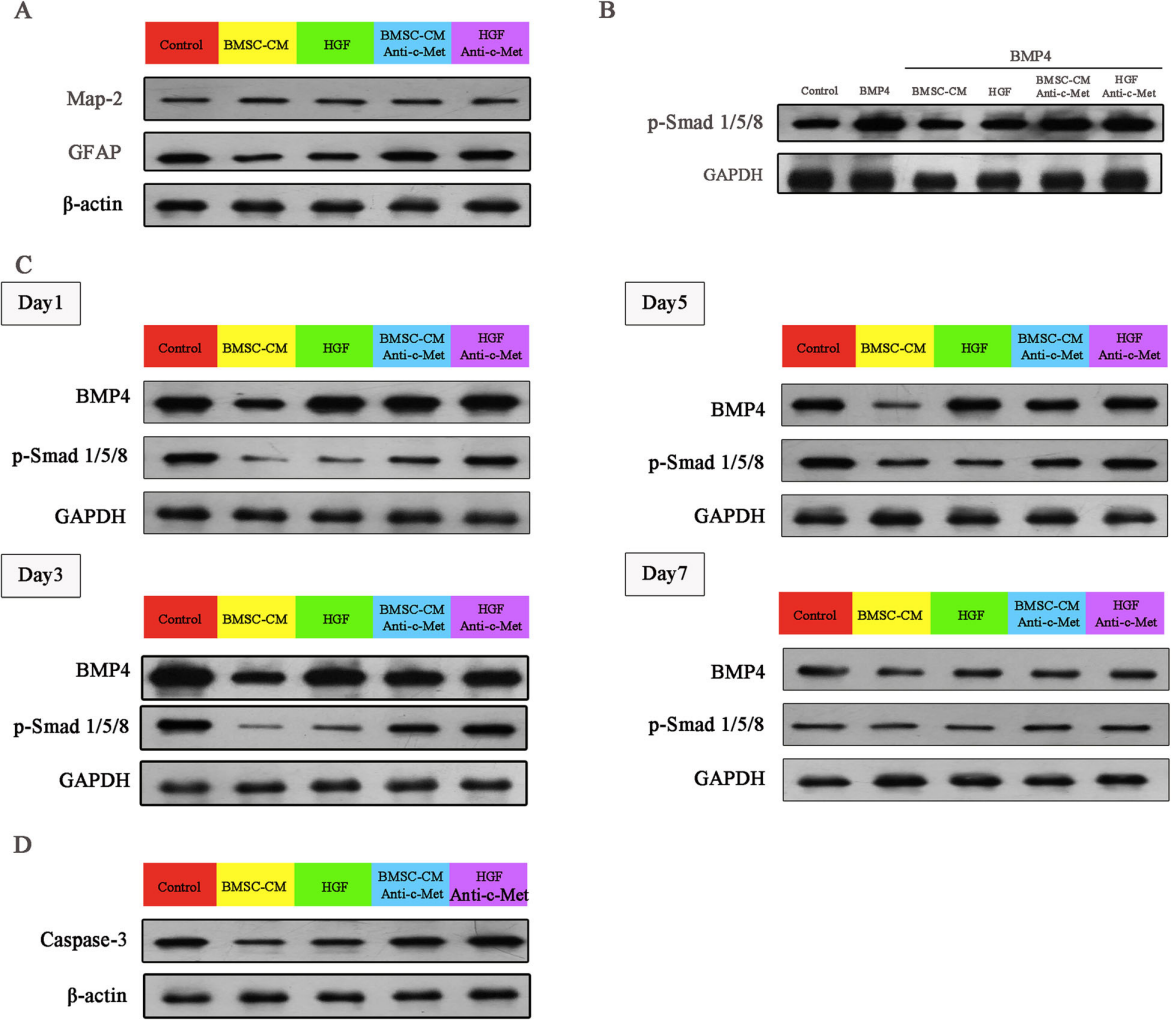
The analysis of protein expression by Western blot. **a** The Map-2 expression and GFAP expression in rats at day 28 after SCI (*n* = 3). **b** The p-Smad 1/5/8 expression in the NSCs (*n* = 3). **c**. The BMP4 and p-Smad 1/5/8 expression in the rats during early time phases of SCI onset (*n* = 3). **d** The Caspase-3 expression in the rats 24 h after SCI onset (*n* = 3)

The biological effect of HGF is modulated via the tyrosine receptor kinase c-Met in many other systems [35–38]. This indicates that the effect of HGF could be negated by the inhibition of c-Met. Therefore, we treated the NSCs with function-blocking c-Met antibodies (50 ng/ml) in the presence of HGF (40 ng/ml). After 7 days of co-culturing, the results showed that blocking c-Met negated the effects of HGF on both the differentiation of the NSCs (Fig. 3a, b) and the modulation of the inflammatory response (Fig. 3e). Compared to the HGF-treated rats, the c-Met antibody-treated rats exhibited lower neuron proportions and higher astrocyte proportions (Fig. 3b) with increasing p-Smad 1/5/8 expression (Figs. 3d and 4c), leading to scar formation around the cavity. The HGF-induced alterations of the inflammatory cytokines were also abolished (Fig. 3e), resulting in the increasing expression of Caspase-3 (Figs. 3g and 4d) and the volume of the cavity (Fig. 3f). As expected, a poor neurological function outcome was also observed in the c-Met pre-treated rats in the presence of HGF (Fig. 3h). All these results proved that the biological effect of HGF is blocked by pre-treatment with the c-Met antibody.

To identify the role of HGF in the BMSC-CM-induced biological effects on SCI rats, we blocked c-Met to assess whether the functional benefits of the BMSC-CM associated with neural differentiation and the inflammatory response can be reversed. With the addition of the c-Met antibodies, the proportion of neurons decreased from 32 to 9%, while the proportion of the GFAP-positive cells markedly increased to approximately 86% after 7 days of co-culturing (Fig. 3i). The increasing expression of p-Smad 1/5/8, induced by the BMSC-CM treatment, was also reduced by the addition of c-Met antibodies (Figs. 3k and 4b). To assess whether the benefits of the BMSC-CM in SCI were also modulated via c-Met, we injected the SCI rats with the c-Met antibodies (200 ng twice per rat, 24 h before BMSC-CM treatment). The rats that received the pre-injection of the c-Met antibodies before the BMSC-CM treatment did not exhibit neurite outgrowth around the cavity (Fig. 3j). Instead, a scar boundary consisting of the GFAP-positive cells was observed around the cavity (Fig. 3j). Western blot also confirmed that the Map-2 expression decreased with the injection of the c-Met antibodies when compared to the BMSC-CM-treated rats (Figs. 3j, 4a). Moreover, the BMSC-CM-induced inhibition of the expression of p-Smad 1/5/8 was also abolished by the pre-injection of the c-Met antibodies in the injured lesion (Figs. 3l and 4c).

Injection with the c-Met antibody also abolished the BMSC-CM-induced effect on alleviating inflammation in SCI rats. We detected the relatively low expression of TNF-α and IL-6 compared to the untreated SCI rats, and these cytokines increased in the rats that received the c-Met antibody treatment (Fig. 3m). Likewise, the IL-4 and IL-10 levels, which increased in the BMSC-CM-treated rats, were reduced after the c-Met antibody treatment (Fig. 3m). Following these alterations, the expression of caspase-3 (Figs. 3o and 4d) and the volume of the cavity (Fig. 3n) also increased and were accompanied by worse BBB scores (Fig. 3p) compared to those of the BMSC-CM-treated rats. All these results proved that HGF played an important role in the biological benefits of BMSC-CM in SCI rats.

### HGF knockdown negated BMSC-CM-induced effects

**Fig. 5:**
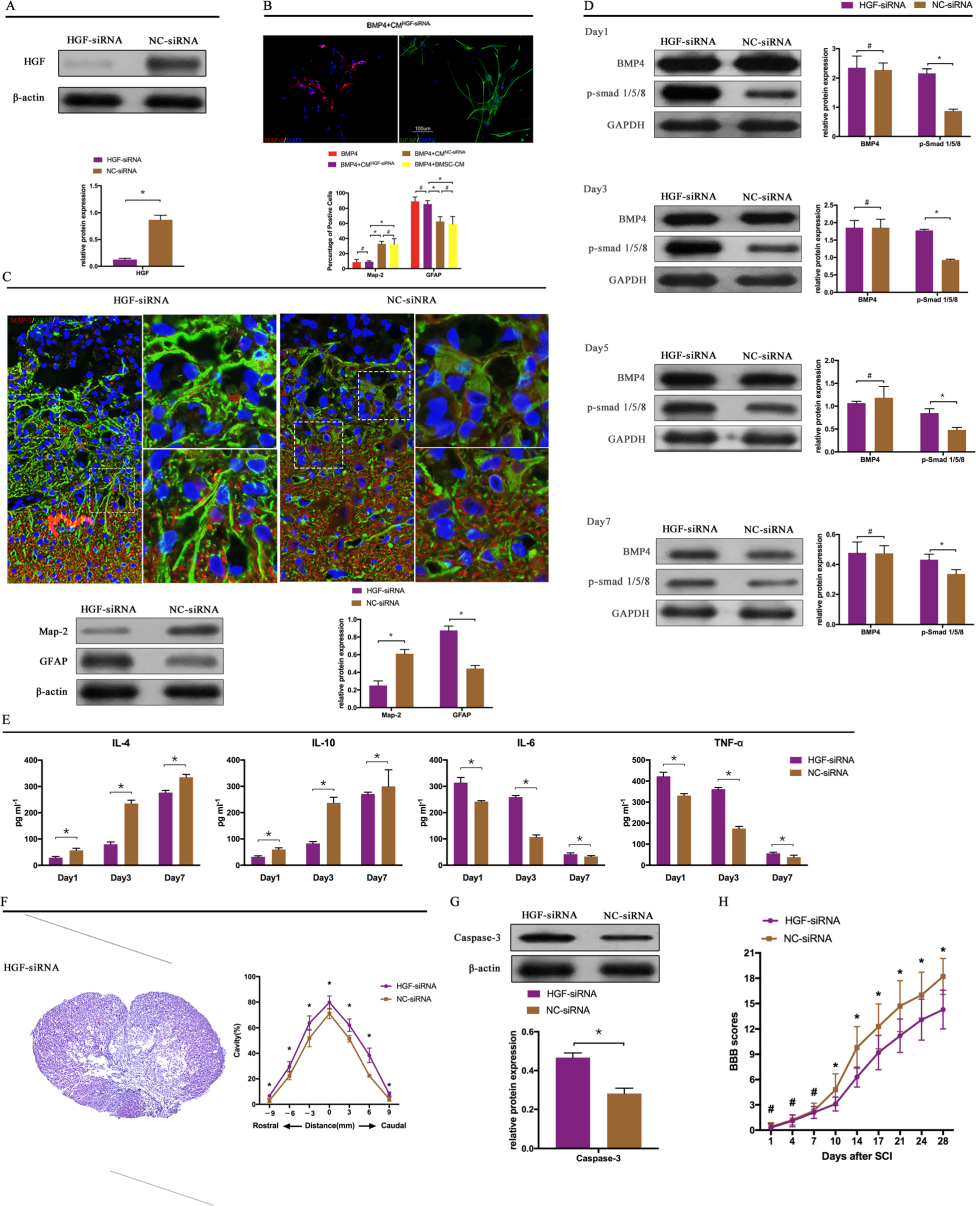
HGF knockdown blocks the BMSC-CM-induced effect. **a** Western blot confirmed that the HGF expression was markedly reduced in BMSCs by the HGF knockdown (*n* = 3). **b** The HGF knockdown made BMSCs lose their ability to mediate the differentiation of NSCs, resulting in a lower expression of neurons and a higher GFAP expression when compared to the BMSC-CM- and CM^NC-siRNA^-treated NSCs in the presence of BMP4 (the percentage of positive cells in BMP4-treated and BMP4 + BMSC-CM-treated NSCs was from Fig. 1a). **c** The treatment of CM^HGF-siRNA^ could not promote neurite outgrowth in the scar bar boundary. This result was also confirmed by Western blot analysis in that the Map-2 expression was reduced in the CM^HGF-siRNA^-treated rats when compared to of the CM^NC-siRNA^-treated rats (*n* = 3). **d** HGF knockdown did not affect the expression of BMP4 (*n* = 3) while it reduced the p-Smad 1/5/8 expression during the early time phases in the SCI rats (*n* = 3). **e** The alterations in the inflammatory cytokines were markedly reduced with the knockdown of HGF in the BMSCs (*n* = 5), resulting in a large cavity volume (**f**, *n* = 3), a higher expression of Caspase-3 (**g**, *n* = 3), and worse BBB score (**h**, *n* = 12). *p < 0.05, ^#^*p* > 0.05; error bars, s.d; scale bars, 100 μm in **a**, **b**; 250 μm in **f**

To further test whether the effect of BMSC-CM was largely dependent on HGF, we used siRNA to knock down the HGF in BMSCS, and the effectiveness of HGF- siRNA was confirmed by monitoring HGF protein levels (Fig. 5a). The CM^HGF-siRNA^ was collected from these BMSCs and concentrated using the same method as for the normal BMSC-CM. To reach the goal, CM^HGF-siRNA^ was used to co-culture with the NSCs for 7 days and then treat the SCI rats. No alterations to the neuron or astrocyte concentrations were found in the CM^HGF-siRNA^-treated NSCs, when compared to the BMP4-alone-treated NSCs. Moreover, the CM^NC-siRNA^-treated NSCs also had a lower proportion of neurons and a higher percentage of astrocytes when compared to the BMSC-CM- and CM^NC-siRNA^-treated NSCs in the presence of BMP4 (Fig. 5b). As expected, the CM^HGF-siRNA^-treated rats exhibited worse neurological outcomes when compared to the CM^NC-siRNA^-treated rats (Fig. 5h). This neurological outcome corresponded to alterations in the spinal cord histology (Fig. 5c). The neurite outgrowth and extension around the cavity was absent in the CM^HGF-siRNA^-treated rats. Similarly, the CM^HGF-siRNA^ partly lost its mediation ability on the inflammatory response (Fig. 5e), resulting in a large cavity volume compared to the CM^NC-siRNA^-treated rats (Fig. 5f). All these results suggested that, without HGF, the BMSCs lost their mediation effect on the neural differentiation and inflammatory response of SCI rats.

## Discussion

ENSCs, which were activated and migrated into the injury lesion following the SCI, have been considered to play a key role in spontaneous self-repair [21, 22, 45]. However, most of these cells differentiated into astrocytes and promoted the scar border formation around the edges of the severely damaged lesion. In the early phase following SCI onset, this scar border is helpful in protecting adjacent surviving neural tissues by limiting the amount of inflammatory cells within areas of the injury lesion [46–48]. Scar over-generation is disadvantageous and had been considered to be the main reason causing the failure of neuron or axon reconnection and regeneration, leading to worse functional recovery [49]. BMPs have been proven to be important factors in the regulation of the differentiation of NSCs. They have been upregulated in the injury lesion following SCI, and this upregulation is associated with the increased expression of astrocytes [19, 50]. BMPs activate their biological function through directly binding to the BMP receptors, which, in turn, induced the phosphorylation of the Smad proteins (Smad1, Smad5, and Smad8). These phosphorylated Smads combine with the common mediator Smad4 and form a heterotrimeric complex. This complex translocates to the cell nucleus and directly or indirectly interacts with the regulation of gene transcription. Through the mediation of these gene transcriptions, BMPs promote the astroglial differentiation of NSCs [22, 51]. Therefore, many studies have focused on the inhibition of the BMP/Smad signaling pathway to attenuate the astrocyte scar border formation. The transplantation of BMSCs is one of the promising therapies for countering the effects of BMPs [22, 38]. It was also confirmed in this study. The BMP/Smad signaling pathway was inhibited by the BMSC-CM treatment and resulted in clear neurite outgrowth and a thinner scar around the cavity.

The post-trauma inflammatory plays a critical role during the entire period of secondary injury and was closely associated with the final extent of neurological deficit [1, 8–10]. Following spinal cord injury, the increasing pro-inflammatory cytokines activate and recruit the inflammatory cells, which have been thought to be a trigger of the inflammatory process. However, in the early phases following the SCI onset, the abnormal over-expression of the pro-inflammatory cytokines accompanied by a low expression of anti-inflammatory cytokines caused excessive inflammation, which contributed to the apoptosis of the nerve cells, an increase in tissue loss, and the enlargement of the cavity. Many studies have revealed that inhibiting inflammation helps reduce the increase in tissue loss and the enlargement of the cavity and promote neurological restoration following SCI [10, 52]. MSCs have been proved to improve neurological function following SCI via modulating inflammation and have been favored by many studies. It was also confirmed by this study. The rats that received the treatment of BMSCs exhibited lower pro-inflammatory cytokine levels and higher anti-inflammatory cytokine levels, accompanied by a reduction in apoptosis protein expression and a smaller cavity volume when compared to the rats without the BMSC treatment.

HGF has not only been found to be a mediator of inflammation in previous studies, but has also been proven to be a neurotrophic factor in the CNS. Kitamura et al. [53] found that, during the early phase following SCI onset, the level of HGF upregulation was significantly less than that of c-Met, suggesting that the injured cord itself is not able to generate a sufficient amount of HGF to support the remarkable increased expression of c-Met. The HGF treatment was able to promote the neuron regrowth, producing a better neurological outcome following SCI. In this study, several results indicated that HGF was crucial in the BMSC-induced biological effects of SCI rats. First, BMSC-CM and HGF had similar effects in terms of improving the neurological function through a combination of neuron regrowth and the mediation of inflammation. Both were able to promote the differentiation of NSCs into neurons. Likewise, in the SCI models, the rats that received either the BMSC-CM or HGF treatments exhibited greater neuron expression, clear neurite outgrowth, and a thinner scar boundary in the injured lesion when compared the rats that did not receive treatment. Furthermore, this study showed that HGF had similar effects on the BMP/Smad signaling pathway as those of BMSC-CM. Previous studies have revealed that HGF is able to counter the capability of BMPs. Kawasaki et al. found that the knockdown of HGF mRNA could upregulate the BMP-2-associated ALP activity in myoblasts. In contrast, this BMP-2-associated activity was inhibited by the exogenous HGF [54]. Similarly, Shibasaki et al. pointed out that c-Met was able to regulate the BMP-2-associated osteoblast differentiation via the PI3K-Akt and Mek-Erk signaling pathways [55]. However, in the CNS, little is known about the relationship between HGF-c-Met signaling and the BMP/Smad signaling pathway. In this study, we proved that the addition of HGF was able to inhibit the phosphorylation of Smad 1/5/8 proteins in vitro and in vivo. Moreover, this HGF-induced effect was completely reversed by the pre-treatment of c-Met antibodies. All these results indicated that HGF had an effect on the differentiation of NSCs via blocking the BMP/Smad signaling pathway. Similarly, the HGF also acted as a suppressor of pro-inflammatory processes. Consistent with previous studies [37, 56], the treatment of HGF significantly downregulated the level of pro-inflammatory factors and upregulated the level of anti-inflammatory factors during the acute phase of SCI, leading to a lower apoptosis of nerve cells and a smaller volume of the cavity. Here, it has been proven that in the immune system, HGF has a similar effect as BMSC-CM.

Secondly, the BMSC-CM-associated effects were abrogated by the inhibition of c-Met in both the CNS and immune system. c-Met, which is mediated by the biological effects of HGF, is expressed in a range of nerve populations, including neurons, oligodendrocytes, astrocytes, and microglia [57]. The stimulation of c-Met promotes neuronal differentiation and axon outgrowth [58], including in SCI rats [53]. Likewise, in the immune system, c-Met has been proven to be expressed in dendritic cells, and HGF was able to promote T cell switching from T_H_1 toward T_H_2 responses [37]. Consistent with this study, the HGF-induced effects were completely reversed by the pre-treatment of c-Met antibodies. Moreover, the effects of BMSC-CM on NSC differentiation and the suppression of the inflammatory response were also markedly diminished by the c-Met antibodies, indicating that the activation of c-Met played a crucial role in the BMSC-CM-exerted biological effects in SCI rats. Finally, we used the CM^HGF-siRNA^ to treat the SCI rats. Only slight improvements in the histology of the injured lesion site and neurological improvement were observed, when compared with the SCI rats.

Based on the results, the biological effect of HGF can be considered concentration-dependent. However, BMSC-CM, with an approximate concentration of 450 pg/ml HGF, was able to exert the same biological effect on the regulation of the differentiation of NSCs as 40 ng/ml HGF alone, the concentration of which was far more than that of BMSC-CM. In addition, the treatment of BMSC-CM was able to decrease both BMP4 and p-Smad 1/5/8 expression, while the treatment of HGF was only able to downregulate p-Smad 1/5/8. This can be explained by the fact that BMSCs release some other growth factors [59, 60], including epidermal growth factor or TNF-alpha. Via a cross-talking, these factors are then able to enhance the biological effect of HGF [61]. BMSCs also secrete some neurotrophins, such as nerve growth factor (NGF) and brain-derived neurotrophic factor (BDNF). These factors have been reported to have a direct relationship with the differentiation of NSCs or the BMP/Smad signaling pathway [61, 62]. This also explained the reason why the pre-treatment with c-Met antibodies or CM^HGF-siRNA^ was unable to affect the expression of BMP4 in the SCI rats.

## Conclusion

BMSC-CM is able to improve neurological function following a SCI by blocking the BMP/Smad signaling pathway to promote NSCs to differentiate into neurons and suppressing the post-trauma inflammatory response. Moreover, these effects might mainly depend on the BMSC released factor: HGF.

## Data Availability

The datasets used and/or analyzed during the current study are available from the corresponding author upon reasonable request.

## Abbreviations

SCI: Spinal cord injury
MSCs: Mesenchymal stem cells
BMSCs: Bone marrow-derived mesenchymal stem cells
NSCs: Neural stem cells
ENSCs: Endogenous neural stem cells
CNS: Central nervous system
TNF-α: Tumor necrosis factor-α
IL: Interleukin
CM: Conditioned medium
BMPs: Bone morphogenetic proteins
HGF: Hepatocyte growth factor
GFAP: Glial fibrillary acidic protein
Map-2: Microtubule-associated protein 2
